# Temporal Sequencing of Multimodal Treatment in Immediate Breast Reconstruction and Implications for Wait Times: A Regional Canadian Cross-Sectional Study

**DOI:** 10.1177/22925503231152261

**Published:** 2023-02-07

**Authors:** Karanvir S. Raman, Maya Morton Ninomiya, Esta S. Bovill, Christopher Doherty, Sheina A. Macadam, Nancy Van Laeken, Kathryn V. Isaac

**Affiliations:** ^1^Department of Surgery, Division of Plastic Surgery, 8166 University of British Columbia, Vancouver, British Columbia, Canada

**Keywords:** breast neoplasms, immediate breast reconstruction, mastectomy, radiation, drug therapy, Cancer du sein, reconstruction mammaire immédiate, mastectomie, radiothérapie, traitement médicamenteux

## Abstract

**Introduction:** Treatment of breast cancer requires a multimodal approach with numerous independent specialists. Immediate breast reconstruction (IBR) adds another layer of coordination to comprehensive breast cancer care. To optimize health outcomes for patients seeking IBR, it is essential to efficiently coordinate the temporal sequence of care modalities inclusive of reconstruction. **Methods:** In this cross-sectional study, patients undergoing IBR following complete or partial mastectomy at one health centre from 2015 to 2021 were included. Patients were categorized into two main groups defined by the first treatment modality received, namely surgery first and Neoadjuvant Chemotherapy. Primary outcome measures were wait times for diagnostic investigations, initiation of treatment, and transitions between therapeutic modalities. **Results:** Of 195 patients, 158 underwent surgery first, and 37 underwent neoadjuvant chemotherapy. Median wait time from first consultation to first treatment initiated in the neoadjuvant cohort was shorter by 11.5 days as compared to the Surgery First cohort (21.5 +/− 19 vs 33.0 +/− 28 days; *P* = 0.001). Twenty-three (82%) of the surgery first and 11 (38%) of the neoadjuvant cohort patients waited longer than 8 weeks for initiation of radiotherapy (*P* = 0.001). Following surgical intervention, the majority of patients failed to meet target benchmarks for transition to chemotherapy (*n* = 25, 53%) and transition to radiotherapy (*n* = 26, 93%; *P* < 0.001). **Conclusion:** Patients undergoing IBR may incur delays in the setting of upfront surgery and in transitioning to adjuvant therapies. In the setting of breast reconstruction, further efforts are required to achieve target wait-times in multimodal breast cancer care.

## Résumé

**Introductıon :** Le traitement du cancer du sein nécessite une approche multimodale faisant intervenir de nombreux spécialistes indépendants. La reconstruction mammaire immédiate (RMI) ajoute une couche supplémentaire de coordination aux soins globaux du cancer du sein. Pour optimiser les résultats sur la santé des patientes souhaitant une RMI, il est essentiel de coordonner efficacement la séquence temporelle des modalités de soins incluant la reconstruction. **Méthodes :** Des patientes ayant subi une RMI après mastectomie complète ou partielle dans un centre de santé de 2015 à 2021 ont été incluses dans cette étude transversale. Les patientes ont été classées dans deux groupes principaux définis par la première modalité thérapeutique reçue : chirurgie d’emblée ou chimiothérapie néoadjuvante. Les critères de jugement principaux étaient les suivants : temps d’attente pour les examens diagnostiques, l’instauration du traitement et les transitions entre modalités thérapeutiques. **Résultats :** Sur 195 patientes, 158 ont subi une chirurgie d’emblée et 37 ont d’abord reçu une chimiothérapie néoadjuvante. Le temps d’attente médian entre la première consultation et le premier traitement instauré a été plus court de 11,5 jours dans la cohorte néoadjuvante par rapport à la cohorte chirurgie d’emblée (21,5 +/- 19 vs 33,0 +/- 28 jours; *P* = 0,001). Vingt-trois patientes (82%) de la cohorte chirurgie d’emblée et 11 patientes (38%) de la cohorte néoadjuvante ont attendu plus de 8 semaines le début de la radiothérapie (*P* = 0,001). Après l’intervention chirurgicale, la majorité des patientes n’a pas pu atteindre les critères cibles de transition vers la chimiothérapie(n = 25, 53%) et la transition vers la radiothérapie (n = 26, 93%; *P* < 0,001). **Conclusion :** Les patientes subissant une RMI peuvent encourir des retards dans l’organisation de la chirurgie d’emblée et la transition vers les traitements adjuvants. Dans le contexte de la reconstruction mammaire, des efforts supplémentaires sont nécessaires pour atteindre les délais d’attente cibles dans le traitement multimodal du cancer du sein.

## Introduction

Breast cancer is the most common cancer in North American women, accounting for up to 30% of new cancer diagnoses in 2020.^1,2^ Comprehensive breast cancer care demands assessment by multiple specialists across the disciplines of Surgical, Medical, and Radiation Oncology.^1,3,4^ Breast reconstruction is an optional component of breast cancer care and has been shown to improve the quality of life in survivorship.^5,6^ For women seeking breast reconstruction, patient management is rendered more complex, with additional considerations for the timing and type of surgery, as well as treatment sequencing.^7^

Delays to treatment may result in adverse health outcomes for breast cancer patients,^3,8^ with breast reconstruction purported as a possible precipitant.^9–14^ Breast reconstruction necessitates additional interventions, both inherently prolonging recovery time and increasing the risk of surgical complications beyond that of ablative therapy alone. As compared to no reconstruction, peri-operative complications are higher with immediate breast reconstruction (IBR) which may lead to re-operation and/or readmission, resulting in potential delays to subsequent therapy.^9,12–14^

The quality of breast cancer care delivery may be evaluated according to wait times.^9–11,13,14^ To date, breast reconstruction patients constitute a minority of the cohorts evaluated. With increasing IBR rates in North America,^15,16^ it is important to assess the delivery of care for breast reconstruction patients to ensure cohort-specific wait time benchmarks are met.^3,4,9,10^

The primary aim of this single centre study was to evaluate and compare the wait times experienced by a cohort of breast cancer patients undergoing immediate reconstruction against Canadian and global standards. Our secondary aim was to determine if neoadjuvant or adjuvant treatments were delayed in this cohort of patients.

## Methods

This cross-sectional study included patients 18 years of age or older undergoing complete or partial mastectomy for therapeutic treatment. Following ablation, all patients underwent IBR with autologous, oncoplastic, or alloplastic procedures by one of five Plastic Surgeons. Autologous reconstruction included the Deep Inferior Epigastric Artery, Transverse Rectus Myocutaneous, and Latissimus Dorsi flaps. Alloplastic reconstruction consisted of either single or two-stage reconstruction with acellular dermal matrix. Oncoplastic procedures included volume displacement or volume replacement techniques with pedicled fasciocutaneous flaps. Patients without a diagnosis of breast cancer who underwent prophylactic mastectomy and reconstruction and/or with missing pertinent data on treatment course were excluded.

Treatments were provided from May 2015 to October 2021. Patient lists were generated by consecutively screening Plastic Surgeons’ medical records for those having undergone IBR. Patient lists were exhausted in reverse chronologic order until target sample of eligible participants had been reviewed. A sample size of 160 patients was required to detect a 20% decrease in the proportion of patients achieving benchmark wait times (alpha 0.05, beta 0.1). Data collection was performed from February 1, 2021, to October 1, 2021, from the date of suspicion, defined as a concerning radiographic screening or physical exam, to either the end of their last treatment or most recent treatment as of October 1, 2021. STROBE Guidelines for cross-sectional studies were followed in the production of this study. Institutional Ethics board approval was obtained.

**Figure 1.**
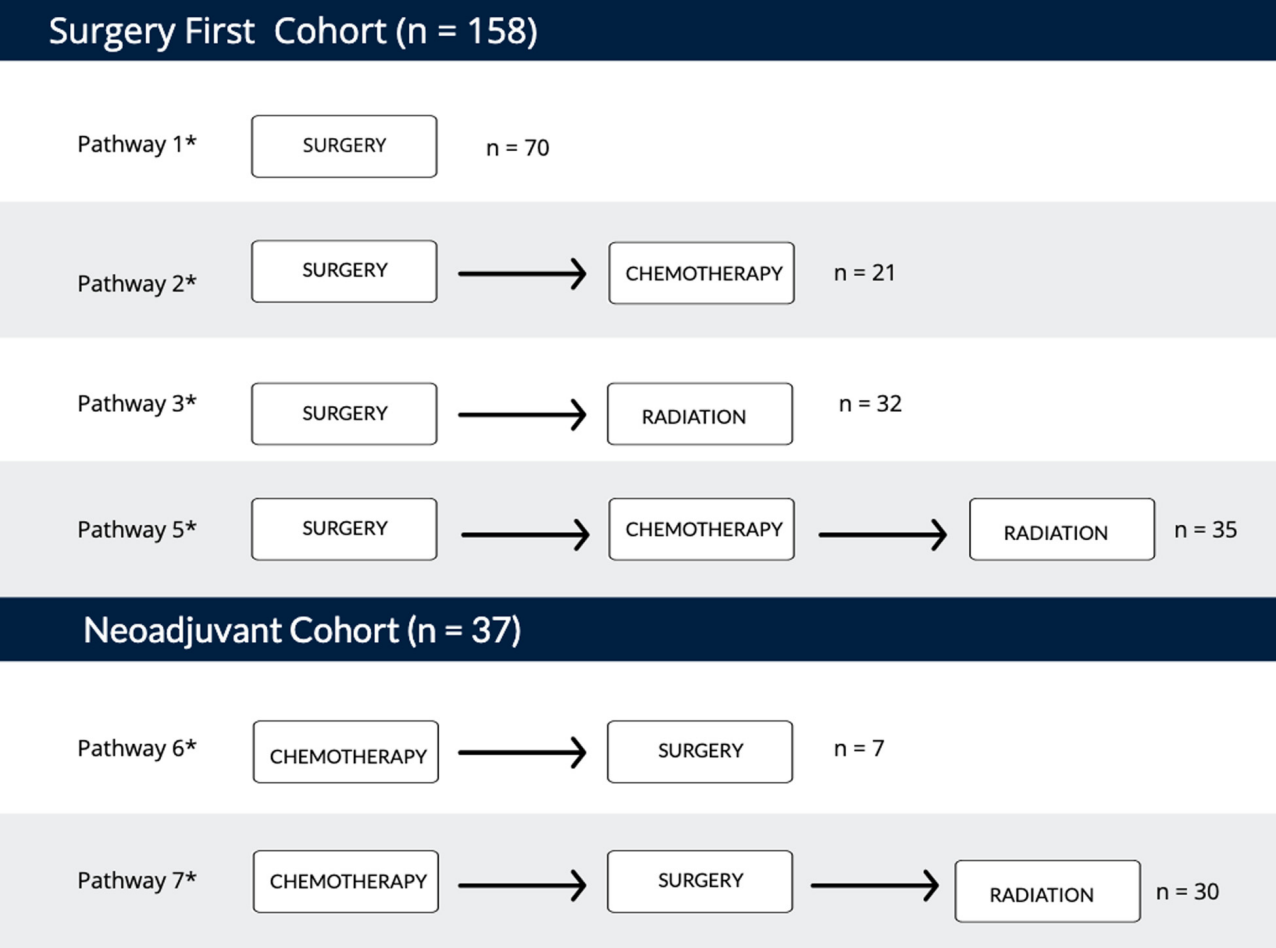
Illustration of care pathways. Order of treatment modalities for eight care pathway variations within surgery first and Neoadjuvant cohorts.

Patients were initially classified according to nine care pathways, defined by the temporal sequencing of treatment modalities (Figure 1). Care pathway cohorts were then categorized into two main groups to examine effects of IBR on treatment delays: (1) Surgery First: undergoing surgical resection and IBR as first treatment; (2) Neoadjuvant: undergoing systemic chemotherapy as their first treatment. Pathways with fewer than five patients were excluded.

*Only care pathways with 5 or more patients were included in the analysis.

Wait times during the pre-treatment and treatment phases were collected and compared for each of the care pathways. In the pre-treatment phase, data was recorded for relevant wait-times between milestone events including: final diagnostic biopsy, referral, consultation, and initiation of first treatment modality. The care process is initiated by the patient's Primary Care Provider. Following patient-reported concerns, clinical exam findings, or abnormal screening mammography, a diagnostic biopsy is obtained. Biopsies in our study consisted of fine needle aspirations or core biopsies. With a positive biopsy finding, the patient is then referred to either Medical Oncology or Surgical Oncology, representing the “first consultation” in our study. “First communication” denotes the documented date a positive biopsy result was provided to the patient.

In the treatment phase, data were collected for wait times for transitions between treatment modalities. Time intervals of interest were defined according to global benchmarks, derived from the Canadian Cancer Society, Canadian Partnership Against Cancer, and European Society of Breast Cancer Specialists (Supplementary Table E1). For time interval measures from Surgery to subsequent therapy, the last surgical date was used to account for bias from surgical complications requiring re-operation, ie, acute re-exploration and re-excision for positive margins. According to the 2019 Pan-Canadian Breast Cancer Surgery guidelines, no more than 10% of patients should wait longer than four weeks from first consultation to initiation of their first treatment.^17^ For radiation therapy (RT), an 8-week interval was established by Huang et Al. for initiation of RT following completion of prior therapy^4^ and the Pan-Canadian standards suggest that no more than 10% of individuals should wait longer than 28 days once ready to treat.^17^ On average, following surgical intervention, patients require an additional 28 days of healing to be considered “ready to treat” with the subsequent modality.^18,19^ Thus, this present study applies the benchmark that no more than 10% of patients should wait longer than 8 weeks or 56 days total following completion of prior therapy. To examine for effects of the COVID-19 pandemic, pre-COVID and post-COVID subgroup analyses were conducted.

Patient characteristics were analyzed using descriptive statistics. Continuous variables are presented as median +/− interquartile range and compared using the Mann–Whitney U test. Categorical measures are presented as proportions and compared using the Fisher exact test. All tests were two-tailed and statistical significance was defined as *p* < 0.05. Statistical analyses were performed with IBM SPSS Statistics, Version 28.0 (Armonk, New York: IBM Corp).

## Results

### Study Population

A total of 213 eligible patients were reviewed; 11 were excluded due to missing data; two were excluded due to being part of a care pathway with fewer than five patients; five were excluded for undergoing only prophylactic surgery. Of the 195 patients included, 158 patients were classified in the Surgery First cohort, and 37 in the Neoadjuvant cohort. Study population and disease characteristics are outlined in Table 1. Pre-diagnostic details are listed in Supplementary Table E2.

**Table 1.** Study Population Characteristics.

	Surgery First			Neoadjuvant	
Characteristic	*n* = 158			*n* = 37	
*Demographic and Risk Factors*					
Age at Diagnosis					
<50 years old	68	43%		24	65%
50–59	42	27%		7	19%
60–69	31	20%		5	14%
70–79	16	10%		1	3%
>80	1	1%		0	0%
BMI (IQR)	23.9	6.3		23.1	5.1
Charlson index (IQR)	3.0	2.0		2.0	2.0
Smoking status					
Never	130	82%		32	86%
Active smoker	9	6%		0	0%
Quit > 1 year ago	16	10%		5	14%
Quit < 1 year ago	3	2%		0	0%
*Diagnosis*					
Laterality					
Unilateral	155	98%		36	97%
Bilateral	3	2%		1	3%
Locally advanced	8	5%		19	51%
Histology^a^					
Invasive carcinoma ductal	89	56%		32	86%
Invasive carcinoma lobular	17	11%		5	14%
Ductal carcinoma in situ	59	37%		0	0%
Lobular carcinoma in situ	2	1%		0	0%
Genetic Markers					
BRCA1/2/PTEN/P53	5	3%		7	19%
Unknown mutation with high risk family history	1	1%		0	0%

*Note:* General characteristics of surgery first versus Neoadjuvant cohorts.

Abbreviations: BMI, body mass index; IQR, interquartile range.

Superscript definitions:

^a^ Some patients in the surgery first cohort had more than one histologic diagnosis on biopsy.

Breast cancer was resected by total mastectomy in 56% (*n* = 89) of surgery first patients and 68% (*n* = 25) of neoadjuvant patients (Supplementary Table E3). Breast conserving surgery (BCS) was performed for the remaining patients, 44% (*n* = 69) for Surgery First and 32% (n = 12) of neoadjuvant patients. In the surgery first cohort undergoing BCS, 15% (*n* = 24) had positive margins requiring subsequent re-excision following BCS, undergoing a median of 1.0 additional procedures. In contrast, only 5% (*n* = 2) of the neoadjuvant cohort had positive margins requiring re-excision following BCS. Of those surgery first patients with positive margins following BCS, 67% (*n* = 16) received a completion mastectomy. Subsequently, 63% (*n* = 10) underwent staged immediate reconstruction with tissue expander (TE) and 13% (*n* = 2) underwent autologous reconstruction. Of the neoadjuvant patients with positive margins following BCS, 50% (*n* = 1) received a completion mastectomy with subsequent autologous reconstruction. For the overall cohort, the majority underwent staged immediate reconstruction with TEs, [*n* = 69 (44%)] for surgery first and [*n* = 20 (54%)] for neoadjuvant. Autologous reconstruction was performed in 16% (*n* = 25) of surgery first and 17% (*n* = 6) of neoadjuvant patients. Oncoplastic reconstruction was performed in 32% (*n* = 51) of surgery first and 30% (*n* = 11) of neoadjuvant patients. Only 8% (*n* = 13) of surgery first patients received a direct to implant reconstruction.

### Care Pathway Comparisons

Median wait-times differed between the care pathways in both the pre-treatment and treatment phases. In the pre-treatment phase, time from final diagnostic biopsy to initiation of first treatment modality was 12.5 days shorter for the Neoadjuvant cohort as compared to Surgery First cohort (48 +/− 38 vs 35.5 +/− 25; *P* < 0.001). Similarly, for first consultation to initiation of first treatment, the neoadjuvant cohort treated 11.5 days sooner than the surgery first cohort (33.0 +/− 28 vs 21.5 +/− 19; *P* = 0.001). For medical oncology referral, time from referral to consult was a median of 7.5 days shorter in the Neoadjuvant group (15.5 +/− 18 vs 8.0 +/− 7; *P* = 0.002).

The surgery first and Neoadjuvant cohorts were evaluated in their transition to subsequent treatment modality. For all surgery first patients, transitions to subsequent treatments were longer. Specifically, the transition from surgery to radiation or chemotherapy (pathways 2 and 3; *n* = 45) (64.0 +/− 30 vs 30 +/− 31; *P* < 0.001) were slower than the Neoadjuvant transition of chemotherapy to surgery (Pathway 6; *n* = 5).

Two pathways include all three treatment modalities: with a sequence of surgery, chemotherapy, radiation (pathway 5; *n* = 29) and sequence of chemotherapy, surgery, radiation (pathway 7; *n* = 29). Time to initiation of RT was compared to assess the influence of the preceding treatment. Patients transitioning from chemotherapy had a shorter median wait time by 8 days (41.0 +/− 21 vs 49.0 +/− 20; *P* = 0.013), suggesting the transition to RT is slower if the preceding treatment is surgery.

Additional intra-pathway comparisons may be found in Table 2.

**Table 2.** Comparison of Care Pathways.

	Surgery First				Neoadjuvant			
Metric	Median Days	IQR	*n*		Median Days	IQR	*n*	*P*
Communication to first Tx	32.0	23	44		29.5	40	6	0.811
first Consult to first Tx	33.0	28	158		21.5	19	32	0.001
Last Bx to first Tx	48.0	38	155		35.5	25	32	<0.001
Last Bx to first Consult	16.0	12	145		12.0	10	35	0.123
Med Onc referral to consult	15.5	18	56		8	7	14	0.002
2 Treatment Modalities^a^								
Transition from first to second Modality	64.0	30	45		30.0	31	5	<0.001
3 Treatment Modalities^b^								
Transition from second to third Modality	41.0	21	29		49.0	20	29	0.013

*Note:* Wait times (median days) between major steps of cancer care pathway for surgery first versus Neoadjuvant cohort.

Abbreviations: Bx,  Biopsy; Tx,  Treatment; Med Onc,  Medical Oncology.

Superscript Definitions:

^a^ Surgery first is inclusive of treatments plans: surgery/chemotherapy and surgery/radiation. Neoadjuvant is inclusive of Chemotherapy/Surgery only.

^b^ Surgery first is inclusive of surgery/chemotherapy/radiation. Neoadjuvant is inclusive of chemotherapy/surgery/radiation.

### Comparison to Benchmarks

#### Initiation of First Treatment

In our surgery first cohort, only 69 (44%) waited fewer than 4 weeks from first consultation to initiation of first treatment. While not meeting the guideline target of 90%, the Neoadjuvant group performed better [*n* = 22 (69%); *P* = 0.012].

#### Adjuvant Treatment Following Surgery

We sought to identify patients waiting longer than 6 weeks for adjuvant treatment following surgery.^4^ Interestingly, 23 (79%) patients in the Neoadjuvant cohort did not meet the 6-week cutoff. This cohort is entirely composed of those receiving chemotherapy, surgery, and then radiation (pathway 7), suggesting a significant delay in transitioning from surgery to RT.

#### Initiation of RT

In the Neoadjuvant cohort, 11 (38%) were waiting longer than 8 weeks, with 5 (17%) waiting longer than 90 days. For those with a delay > 6 weeks and < 90 days, 15 (83%) had received an RT consultation prior to surgery (Table 4).

**Table 3.** Wait Times Relative to Benchmarks.

	Surgery First				Neoadjuvant				
	Total	Failed to Meet Benchmark			Total	Failed to Meet Benchmark			
Benchmark	*n*	*n*	%		*n*	*n*	%		*P*
> 6 weeks from Dx to first Tx	156	93	60%		32	10	31%		0.006
> 4 weeks from first consult to first Tx	158	89	56%		32	10	31%		0.012
> 6 weeks from surgery to second Tx	75	51	68%		29	23	79%		0.087
Second Tx RT	28	26	93%		29	23	79%		0.253
Second Tx Chemo	47	25	53%						<0.001^a^
> 8 weeks for RT initiation	57	28	49%		29	11	38%		0.366
From surgery to RT	28	23	82%		29	11	38%		0.001
From chemo to RT	29	5	17%						<0.001^b^
> 90 days for RT initiation	57	7	12%		29	5	17%		0.528
From surgery to RT	28	5	18%		29	5	17%		1.000
From chemo to RT	29	2	7%						0.253^c^
> 90 days for chemo initiation	47	2	4%		32	1	3%		1.000

*Note*: Percentage of surgery first and Neoadjuvant cohorts failing to achieve benchmark wait times. *P* values comparing proportion of those failing to meet benchmark.

Abbreviations: RT, radiation therapy; Tx, treatment; Chemo, chemotherapy; Dx, diagnosis, defined as date of last biopsy; d, days.

Superscript Definitions:

^a^ Comparison of time from surgery to second treatment: Surgery to radiation [*n* = 26 (93%)] versus surgery to chemotherapy [*n* = 25 (53%)].

^b^ Comparison of time to transition to radiotherapy: Surgery to radiation [*n* = 23 (82%)] versus chemotherapy to radiation [*n* = 5 (17%)].

^c^ Comparison of time to transition to radiotherapy: Surgery to radiation [*n* = 5 (18%)] versus chemotherapy to radiation [*n* = 2 (7%)].

**Table 4.** Delay to Initiation of Radiation Therapy.

	Surgery→Radiation			Chemotherapy→Surgery→Radiation	
	Delay 6 weeks to 90 days	Delay > 90 days		Delay 6 weeks to 90 days	Delay > 90 days
	*N* = 21	*N* = 5		*N* = 18	*N* = 5
RT consult pre-operative	1 (5%)	1 (20%)		15 (83%)	1 (20%)
RT consult post-operative	20 (95%)	4 (80%)		3 (17%)	4 (80%)
**Reasons for Delay:**					
Timing of RT referral^a^	15 (71%)	5 (100%)		7 (39%)	3 (60%)
Management discussion^b^	11 (52%)	3 (60%)		6 (34%)	5 (100%)
Plastic surgery related^c^	3 (14%)	0		5 (28%)	0
Adjuvant chemotherapy^d^	0	0		12 (67%)	2 (40%)
Patient preference^e^	0	4 (80%)		0	2 (40%)

*Note*: Explanatory factors for a prolonged delay to RT initiation in the surgery first and Neoadjuvant groups. Patients may have more than one identifiable cause for delay in radiation therapy.

Abbreviations: RT, = radiation therapy; d, days; wk, weeks

Superscript:

^a^ Timing of referral contributed to delay

^b^ Ongoing multi-disciplinary discussion regarding further management (RT indications, additional chemotherapy, or staging procedures),

^c^ Plastic surgery-related complications and/or delay for TE expansion

^d^ Patient receiving additional post-operative chemotherapy

^e^ Patients non-compliant with therapy or personal preference for delayed therapy

For those undergoing surgery first, 23 (82%), waited longer than 8 weeks, significantly longer than the 11 (38%) patients in the Neoadjuvant cohort (*P* = 0.001). This difference was not present at the 90-day assessment [*n* = 5 (7%) versus *n* = 5 (17%); *P* = 1.000)]. For delays > 6 weeks and < 90 days in this surgery first cohort (*n* = 21), the majority, 20 (95%) had received a RT consult after surgery (Table 4).

Of those transitioning from surgery to chemotherapy and concluding with RT, 24 (83%) patients transitioned to RT within 8 weeks of concluding chemotherapy. This is significantly better than those undergoing only surgery with subsequent RT [*n* = 5 (18%); *P* < 0.001]. While this difference resolves by the 90-day point [*n* = 50 (82%) versus *n* = 23 (93%); *P* = 0.253], the preliminary transition to RT is slower if preceded by surgery.

#### Transition Following Surgery

Transitions from surgery to either chemotherapy or radiation were evaluated against the 6-week interval benchmark for subsequent treatment. Of those transitioning from surgery to chemotherapy, 25 (53%) patients failed to meet the 6-week cutoff. Wait times were significantly worse for those transitioning from surgery to RT [*n* = 26 (93%); *P* < 0.001], suggesting that following surgery, the transition to RT is slower than to chemotherapy.

#### Initiation of Chemotherapy

For chemotherapy initiation, no more than 10% of patients should wait longer than 90 days following completion of prior therapy or consultation with medical oncology.^3,9,10,17^ All patients met this benchmark, including those in both care pathways receiving chemotherapy following surgery (pathways 2 and 5) and those receiving chemotherapy first following consultation (pathways 6 and 7).

Additional comparisons to benchmarks may be found in Table 3.

## Discussion

### Summary of Findings

In this single centre study, patients undergoing surgery first with resection and IBR experienced longer delays to their first treatment following consultation or final diagnostic biopsy when compared to patients receiving Neoadjuvant systemic treatment. The transition from surgery to RT is delayed, particularly if the patient is undergoing surgery as their first treatment modality, as compared to those receiving Neoadjuvant systemic treatment. Patients undergoing surgery first take longer to transition to the subsequent treatment modality than the Neoadjuvant Cohort. Following surgery, the transition to chemotherapy is more efficient than the transition to RT.

### Time to First Treatment

Only 44% of the surgery first cohort in our study met the 2019 Pan-Canadian guidelines advocating for a delay no longer than 4 weeks from first consultation to first treatment.^17^ In this group, median time from first consultation with medical or surgical oncology to first treatment was 33 days; subgroup analysis of first consultation with surgical oncology to surgery as first treatment was 32 days. While the wait times are acceptable in relationship to the benchmark 4 weeks,^17^ they are longer when compared to prior studies. Cha et al.^20^ demonstrate a delay of 24 days with 24% of patients undergoing IBR and Cadili et al.^21^ report a 23-day delay with 15% undergoing IBR. In contrast to these two cohort studies, our present study is exclusively patients undergoing IBR, adding complexity known to increase delays.^9–14^ Our study population necessitates an additional provider consultation with the plastic and reconstructive surgeon. Furthermore, our definition for “first consultation” is broader including not only surgical oncology, but also medical oncology. Given that we do not have a comparable cohort not undergoing IBR, we cannot conclusively quantify the effects of IBR. Rather, we have compared wait times to standardized benchmarks and demonstrate that patients desiring IBR experience longer wait times.

For those undergoing surgery first, patients were slower to receive first treatment modality following their first consultation by 11.5 days as compared to those receiving Neoadjuvant systemic therapy first. This may result from the need to coordinate surgical therapy, with additional surgical consultation and operative time. Consultation with a Plastic Surgeon has been previously reported to delay therapy by up to 10 additional days.^11,22,23^ Imaging to assist in surgical planning, such as an MRI, contributes upwards of 6 days of delay^24,25^ and may inadvertently increase the number of subsequent pre-operative investigations and consultations, further delaying time to Surgery.^22–26^ Moreover, operative resources for IBR are a specific and scarce resource in Canada^27^ whereas an infusion clinic for chemotherapy has greater capacity,^28–30^ is supported by allied health care professionals, and is thus likely more readily mobilized.

### Initiation of Radiotherapy

Fewer than 90% of patients started RT within 8 weeks of concluding either surgery or chemotherapy. Congruent with previous reports,^31^ there is a stark improvement in efficiency if a patient is in a care pathway which entails receiving Neoadjuvant systemic therapy upfront. Despite this finding, our results must be interpreted within the context of the study design. With chemotherapy being delivered over the course of several weeks, this affords adjunctive care providers the time to perform consultations, preemptively plan, and arrange for subsequent therapy such as Radiation. In fact, the confounding nature of an earlier consultation with radiation oncology being facilitated by medical oncology was explored in our study. Specifically, with the surgery first cohort proceeding directly to RT, 95% of RT consultations occurred post-operatively. In contrast, 83% of patients undergoing Neoadjuvant chemotherapy received an opinion regarding RT prior to surgical intervention. This early consultation is typically initiated by medical oncology and allows for efficient transition in therapeutic modalities. The delay to RT consultation may also be explained by disease characteristics, discussed further below. Half of those undergoing Surgery First and proceeding to RT (without chemotherapy) have less advanced disease. This cohort waits a median of 16 days for review of pathology to then have RT referral initiated, which may have a perceived reduced urgency for initiation of RT.^31^

For patients receiving all three treatment modalities, our study suggests the delay in transitioning to RT is worse if preceded directly by Surgery, as opposed to Chemotherapy. We would suspect this is secondary to the inherent predictability and efficiencies in delivering Chemotherapy, with predefined conclusion dates and lack of surgical complications delaying radiation. Moreover, the co-location of providers may influence the delay. Synergies of a multidisciplinary clinic (MDC) decrease wait times to consultation and time to therapy.^32–37^ MDCs increase adherence to standardized guidelines, patient satisfaction, and rates of reconstruction following mastectomy.^11,37–42^

### Transition from Surgery

Transition from Surgery to Chemotherapy is notably shorter than the transition to RT. Furthermore, our surgical date is inclusive of complications requiring re-exploration and re-excision for positive margins, thus excluding these as explanatory variables. Following Surgery, patients would arguably undergo a similar post-operative sequence of events, yet chemotherapy is mobilized faster. Analogous to OR time, RT capacity has not grown in proportion to the demand for RT.^31^ Further evaluation is required to elucidate resource limitations and other causative factors.

### Implications

Ultimately, wait times must be judged contextually in relation to both histopathologic status and health outcomes. For non-invasive cancers, there is less urgency in shared decision making given the minimal or insignificant likelihood of adverse health outcomes with delay.^43^ In contrast, patients with advanced stage cancer are more likely to experience worse outcomes if the delay from diagnosis to first treatment is longer than 60 days^44^ or if delay to Chemotherapy following surgery is greater than 61 days.^45^

In our study, those experiencing a longer delay to consultation with Medical Oncology were also lower risk. Time to consultation with Medical Oncology in the Neoadjuvant cohort was almost twice as fast as compared to the Surgery First cohort, which was compromised of 33% of patients with in situ disease. For patients with DCIS or even for patients with stage 1 invasive carcinomas, studies have shown that modest delays in time to surgery will not significantly impact treatment outcomes.^43^ Thus, patients may be reassured that their clinical outcomes will not be immediately compromised due to a modest delay and take the time needed to be involved in the decision-making process surrounding their surgery.^43^

Post-mastectomy RT, an integral component of breast cancer therapy, was delayed in our cohort. As previously mentioned, transition to RT following Surgery exhibits the largest delays, with 82% of patients (pathway 3, surgery first) waiting longer than 8 weeks.^4^ Delays in our cohort may be explained by certain patient characteristics. Although all patients in pathway 3 underwent RT following surgery, half were diagnosed with a non-invasive malignancy. Carrying a more favorable prognosis, delays up to and longer than 8 weeks do not necessarily confer a difference in recurrence and may indeed be tolerable with appropriate reassurance.^46,47^ However, this does not hold true for the remaining pathways including RT, with all constituent patients’ having a nearly exclusively invasive diseases diagnosis. In which case longer delays may be associated with an increase in local recurrence,^47–49^ with nearly a two-fold increase from 5.8% to 9.1% for waits longer than 8 weeks.^4^ This recurrence rate translates into hundreds of new cases per year at a national level and may prove to be determinantal to the overall efficacy of an RT program.^47,48^ Our study findings are in line with the scarcity and lack of adequate growth in RT capacity in Canada.^28,31^ In addition, prolonged post-operative complications associated with IBR may certainly contribute in delays to subsequent RT. However, we would expect the delays to be equally pronounced in both the Chemotherapy and RT groups. A comparison control group of patients not undergoing reconstruction would help elucidate a causal relationship between reconstructive procedures and delays to subsequent therapies.

### Limitations

Given the geographic limitations of our study population, generalizability of these results may be limited to other regional jurisdictions. While the strength of our paper is its focus on IBR patients, our findings are not applicable to larger population-based studies of breast cancer patients which include a minority undergoing IBR. Lastly, our study dates are inclusive of the COVID-19 Pandemic which reduced the overall number of eligible patients undergoing IBR.

## Conclusion

Wait times for breast reconstruction patients are prolonged when transitioning between treatment modalities. Specifically, patients undergoing IBR may incur delays in the setting of upfront surgery and in transitioning to adjuvant therapies. In the setting of breast reconstruction, further resources are required to achieve target wait times in multimodal breast cancer care.

## Supplemental Material

sj-docx-1-psg-10.1177_22925503231152261 - Supplemental material for Temporal Sequencing of Multimodal Treatment in Immediate Breast Reconstruction and Implications for Wait Times: A Regional Canadian Cross-Sectional Study Supplemental material, sj-docx-1-psg-10.1177_22925503231152261 for Temporal Sequencing of Multimodal Treatment in Immediate Breast Reconstruction and Implications for Wait Times: A Regional Canadian Cross-Sectional Study by Karanvir S. Raman, MD, BBA, Maya Morton Ninomiya, Esta S. Bovill, MD, PhD, Christopher Doherty, MD, MPH, Sheina A. Macadam, MD, MSH, Nancy Van Laeken, MD, and Kathryn V. Isaac, MD, MPH in Plastic Surgery
