# Supplementary material for: Temporal Sequencing of Multimodal Treatment in Immediate Breast Reconstruction and Implications for Wait Times: A Regional Canadian Cross-Sectional Study
Source: Plast Surg (Oakv). 2023 Feb 7;32(4):583–92. doi: 10.1177/22925503231152261 (PMC11489937; doi:10.1177/22925503231152261)
Supplement: sj-docx-1-psg-10.1177_22925503231152261 - Supplemental material for Temporal Sequencing of Multimodal Treatment in Immediate Breast Reconstruction and Implications for Wait Times: A Regional Canadian Cross-Sectional Study [file sj-docx-1-psg-10.1177_22925503231152261.docx]

# Supplementary Table E1 – Definition of Benchmarks

| **Metric** | **Recommended Guidelines** |
| --- | --- |
| > 2 weeks from medical oncologist referral to consult | According to the 2019 Pan Canadian Standards it is recommend that in the case of neoadjuvant therapy patients should be assessed by medical oncology within two weeks of referral^12^. |
| > 6 weeks from diagnosis to 1st Treatment | The European Society of Breast Cancer Specialists recommends that patients undergo surgery within 6 weeks of 1st diagnostic imaging[^17^](https://paperpile.com/c/kdFMnT/MJ1E). |
| > 4 weeks from 1st consult to 1st Treatment | According to the 2019 Pan Canadian Standards, the initial treatment (either surgery, systemic therapy and/or radiation therapy) should be initiated within 4 weeks of consultation[^12^](https://paperpile.com/c/kdFMnT/gpmz). |
| Delay > 8 weeks for Radiotherapy (RT) initiation | A systematic review by Huang et al., recommends patients receive postoperative RT no more than 8 weeks after surgery^3^. |
| Delay > 90 days for chemotherapy initiation | Retrospective analyses recommend initiation of adjuvant chemotherapy within approximately 84-90 days following surgery^2, 10, 11, 12^. |

# Supplementary Table E2 – Pre-Diagnostic Investigations

|  | **Surgery First** | |  | **Neoadjuvant** | |
| --- | --- | --- | --- | --- | --- |
| **Characteristic** | **n = 158** | |  | **n = 37** | |
| **Pre-Diagnosis** |  |  |  |  |  |
| **Detection Type**^1^ |  |  |  |  |  |
| Mass Self Detected | 67 | 42% |  | 29 | 78% |
| Mass Clinical Exam | 8 | 5% |  | 1 | 3% |
| Screening imaging | 66 | 42% |  | 3 | 8% |
| Other^2^ | 14 | 9% |  | 2 | 5% |
| Discharge | 5 | 3% |  |  |  |
| Indentation/Indrawing | 3 | 2% |  | 1 | 3% |
| Dry Skin/Rash | 3 | 2% |  |  |  |
| Number of Biopsies (IQR) | 1.0 | 0.0 |  | 1.0 | 0.0 |
| Number of Imaging (IQR) | 3.0 | 2.0 |  | 3.0 | 1.0 |
| Days from Screening to 1st Diagnostic Test (IQR) | 9.5 | 10.3 |  | 15.0 | 4.5 |

*Abbreviations: IQR = Interquartile Range*

Superscript:

1. Data available for 155/158 Surgery First and 35/37 Neoadjuvant patients
2. Three most common “other” causes are provided.

# Supplementary Table E3 – Surgical Interventions

|  | **Surgery First** | |  | **Neoadjuvant** | |
| --- | --- | --- | --- | --- | --- |
| **Characteristic** | **n = 158** | |  | **n = 37** | |
| **Tumor Resection** |  |  |  |  |  |
| Total Mastectomy | 89 | 56% |  | 25 | 68% |
| Partial Mastectomy | 69 | 44% |  | 12 | 32% |
| **Repeat Surgery for Positive Margins** |  |  |  |  |  |
| Positive Breast Margins after BCS | 24 | 15% |  | 2 | 5% |
| Partial Mastectomy as Repeat Surgery^1^ | 16 | 10% |  | 2 | 5% |
| Completion Mastectomy as Repeat Surgery | 16 | 10% |  | 1 | 3% |
| Repeat Breast Procedures for Positive Margins (median, IQR) | 1.0 | 0.0 |  | 1.5 | 0.5 |
|  |  |  |  |  |  |
| **Breast Reconstruction** |  |  |  |  |  |
| Tissue Expander | 69 | 44% |  | 20 | 54% |
| Direct To Implant | 13 | 8% |  | 0 | 0% |
| Latissimus Dorsi | 4 | 3% |  | 1 | 3% |
| DIEP | 13 | 8% |  | 5 | 14% |
| TRAM | 8 | 5% |  | 0 | 0% |
| Oncoplastic | 51 | 32% |  | 11 | 30% |
| Days from Referral to Consultation (median, IQR) | 11.0 | 12.0 |  | 20.0 | 23.0 |

*Abbreviations: DIEP = Deep Inferior Epigastric Perforator Flap; TRAM = Transverse Rectus Abdominis Myocutaneous, IQR = Interquartile Range*

*Superscript:*

1. Some patients with positive margins underwent more than 1 repeat surgical resection
